# A Study on the Construction and Validation of Pathways to Sustainable Participation in Outdoor Activities Among Chinese Elderly Individuals

**DOI:** 10.3390/ejihpe15070116

**Published:** 2025-06-20

**Authors:** Jia Wei, Mohd Johari Mohd Yusof, Shureen Faris Abdul Shukor

**Affiliations:** Faculty of Design and Architecture, Universiti Putra Malaysia, Serdang 43400, Selangor, Malaysia; shureen@upm.edu.my

**Keywords:** transtheoretical model (TTM), environmental psychology, confirmatory factor analysis, Chengdu City, senior outdoor survey (SOS) tool, community attachment measurement (CAM)

## Abstract

The objective of this study was to propose an analysis pathway that illustrates the psychological mechanism and corresponding environmental motivators affecting the frequency and duration of outdoor activity participation among older adults in China. Firstly, based on the transtheoretical model (TTM) theory and the environmental psychological approach, a literature review was conducted to select the model variables and ensure that they correspond to the requirements of the stage of change construct and the temporal dimension of the TTM theory. Secondly, the variables mentioned above were rearranged according to the perception, action, experience, and emotion stages of the individual psychological mechanism process and the environmental quality improvement outcome to develop the hypothetical model. Subsequently, a confirmatory factor analysis was carried out to test the hypotheses and validate the model based on the survey data collected in Chengdu City, China. Finally, a total of 372 valid questionnaires were received. After analyzing the collected data, the configuration index relationship of the hypothesis model was validated. In conclusion: Through slicing control of environmental factors in diverse human-environmental interaction stages under the guidance of the stage-based behavioral analysis discipline, this study explores an analysis pathway for upgrading outdoor environment facilities to enhance the attraction and attachment characteristics of the environment and, in turn, promote the sustainability of the outdoor activities performed by older people.

## 1. Introduction

The outdoor activities of older people are influenced by a variety of factors at macro-, meso-, and micro-levels, as well as by interdisciplinary research perspectives. For instance, studies in the discipline of individual behavioral promotion (Nigg & Durand, 2016; Scherrens et al., 2018) and macro public management and policy (Mercader-Moyano et al., 2020). Kilgour et al. (2024) concludes that “Identifying common barriers and motivators for older adults is a key step in the development of strategies to promote PA.” Normally, there are two types of barriers, personal and environmental. Personal barriers include such things as one’s level of motivation, physical capabilities amount of leisure time available, financial resources, and the extent of family, work, and household obligations. Environmental barriers are obstacles that are imposed by the built environment and, occasionally, the natural environment (e.g., the weather) (Frank et al., 2003). These barriers include the availability of facilities for different types of physical activity, the distance between home and such facilities or other destinations, and the perceived quality and safety of the environment in which one wishes to participate in a certain activity. Motivators can also adapt by erasing the above barriers.

McMullan et al. (2021) conclude there is a need for designing effective and sustainable interventions to promote PA in the long term in older adults for it has large beneficial effects on the elderly health. In summary, Warburton (2006) confirms that there is irrefutable evidence of the effectiveness of regular physical activity in the primary and secondary prevention of several chronic diseases (e.g., cardiovascular disease, diabetes, cancer, hypertension, obesity, depression, and osteoporosis) and premature death. Vogel et al. (2009) finds that physical activity is linked to a reduced risk of developing dementia and Alzheimer’s disease. Hu et al. (2023) conclude the engagement of immigrants in recreational outdoor activities is an indicator of community engagement leading to social benefits, increasing interactions across diverse groups, and making a sense of belonging to one’s community.

Many studies analyze and dissect the motivators to promote physical activities strictly from the spatial perspective, neglecting the temporal dimension. In other words, they assess an individual’s readiness to participate in activities as influenced only by physical spatial motivators (Lin, 2017), but overlook the duration of the influence and its significance regarding the sustainability of participation. McMullan et al. (2021) conclude ways of ensuring effective maintenance of physical activity levels in older adults are unclear. De Jesus et al. (2017) conclude there is a need for designing effective and sustainable interventions to promote PA in the long term in older adults. In fact, according to Gehl’s three activity laws concerning the environment and public behaviors (Gehl, 2002), the process of activity participation also generates attraction and promotes the sustainability of activity occurrence. This implies that the duration of time is another mediating variable that cannot be overlooked when analyzing how environmental motivators promote participation in outdoor activities.

Therefore, this study aims to enhance the understanding of the impact of participation in leisure activeities by older people and its effects on the human–environment interaction process. As the factors promoting behavior should be considered in both spatial and temporal dimensions, this study adopts the TTM theory as its core theoretical framework, emphasizing processability and maintenance. More specifically, this study focuses on the dynamic psychological changes occurring in the interaction process between individuals and their environment. As the research focuses on environmental motivators, environmental psychology was chosen as the other theoretical basis.

## 2. Theoretical Basis and Research Hypotheses

### 2.1. TTM

As a behavioral change theory, the transtheoretical model has been widely used as a tool to facilitate specific behavioral changes (Rhodes et al., 2021, p. 17). It includes the following main constructs: (1) stage of change—intention to take action; (2) decisional balance—pros and cons associated with a behavior’s consequences; (3) self-efficacy—confidence to make and sustain changes in difficult situations; (4) processes of change—ten cognitive, affective, and behavioral activities that facilitate change (Prochaska et al., 2005). Some studies also include (5) situational temptation (Scherrens et al., 2018, p. 17). These constructs can be explored, operationalized, or measured to define the structural and psychological determinants of specific behaviors (Scherrens et al., 2018, p. 3); for instance, Prochaska et al. (2005) proposed that “Individuals in the Action and Maintenance stages report higher self-efficacy than those in the Contemplation and Preparation stages”, and that “individuals weigh the cons of being emotionally ready as higher than the pros, while the opposite is true in the Maintenance stage”.

### 2.2. Environmental Psychology

Stage-based psychological indicator measures based on the TTM can provide sensitive assessments of emotional readiness to initiate and maintain a behavior or activity. Therefore, “One of the initial challenges is identifying criteria that represent appropriate stages” (Prochaska et al., 2005, p. 141). For this reason, environmental psychology was chosen as the other theoretical basis. Russell and Ward (1982) have defined environmental psychology as a branch of psychology that is concerned with providing a systematic account of the relationship between a person and their environment. Furthermore, the locational specificity of behaviors is a fundamental fact of environmental psychology.

### 2.3. Reinterpreting Core Constructs and Developing a Hypothetical Model

The construction of the research model is based on stage-based environment perception and cognition psychological variables, which are used as the model configuration indicators. The TTM describes the general psychological process for behavior change, but certain application situations deserve special discussion. This study focuses not on psychological perspectives for individual behavior change but on motivators belonging to environmental perspectives. Therefore, reinterpreting core constructs in connection to environment perception and cognition measurement is the first step in applying the TTM to construct the research model.

Although the TTM theory contains several sub-theories (Nigg & Durand, 2016), it systematically integrates four theoretical constructs that are central to change (Prochaska et al., 2005). This study mainly refers to the stages of the change construct. First and foremost, the temporal dimension is the basis and core essence of the stages of change construct. Conferred to this construct, two different concepts are employed to divide behaviors into pre-action and post-action (Prochaska et al., 2005). Before the target behavior change occurs, the temporal dimension is conceptualized in terms of behavioral intention. After the behavioral change has occurred, the temporal dimension is conceptualized in terms of the duration of the behavior (Velicer et al., 1998). Raymond et al. (2010) have proposed that individuals in the early stages rely more on cognitive, affective, and evaluative processes of change; while individuals in the later stages rely more on social support, commitments, and behavior management techniques. This provides theoretical guidance for proposing the “attraction” and “attachment” environmental measurements as the configuration of outcome variables.

Attraction here mainly refers to the stage of the psychological process before taking action. According to the TTM theory, people go through at least three stages before action, including emotional attraction at the perceptual level. Then, the pre-contemplation and contemplation stages involve considering the pros and cons of the behavior (Velicer et al., 1998). In reality, individuals remain attracted due to the environmental interaction behavior values before the activity. When this attraction is strong enough, a significant driving force will be generated to drive people to take action.

After taking action, the focus of consideration shifts to the maintenance stage of the transtheoretical model (TTM). Maintenance is related to the attachment notion pertaining to social psychology in a sense. According to attachment theory, it refers to the process of a person’s attachment to an object, which can be a tangible entity such as an animal, product, or environment (Casakin et al., 2021), or an abstract concept, such as brand attachment (Park et al., 2006). The research subject is the aging community outdoor environment, thus, the primary consideration is community attachment theory (Ma, 2021). Lin (2017) found that a sense of community may affect the community participation of residents, reflecting emotional attachment in the ability of places to become settings for daily activities.

In accordance with the TTM theory’s stage of change construct, the temporal dimension’s two stages are further manifested through five psychological stages, indicating the stages in which people perceive the environment and react when they interact with it. Therefore, the development of measures for the core five variable constructs is the second step in applying the TTM. Different from the original research object in the TTM theory for behavior, this study focuses on interactions between the environment and humans. Thus, there is an adaptation when considering the five stages which emphasize the environment and human interaction while neglecting other discipline perspective influencement. The adapted five stages are “Perception”, “Impression”, “Preparation”, “Action”, and “Maintenance”, and the corresponding measurements are proposed below.

1. “Perception” stage—Visibility

Level one is the awareness of the surrounding environment. According to landscape presentation and sensory dimensions of landscape perception, people perceive the environment primarily through the visual system (the visual sense), gustatory system (the sense of taste), olfactory system (the sense of smell), vestibular system (the sense of balance), kinesthetic system (the ability to sense and coordinate movement), tactile system (the sense of touch), and auditive system (the sense of hearing) (Roy et al., 2021; Collier & Jakob, 2017). Bruce et al. (2014) found that 87% of human perception is based on sight. Moreover, 80% of our impressions of our surroundings come from sight. As the elderly age, their vision significantly declines. Therefore, the visibility of environmental facility design is crucial and set as the configuration indicator of this perception stage. In fact, it is not only a vital source of environmental perception but also a key basis extending to the whole human and environment interaction stages.

2. “Impression” stage—Invitingness

Level two emerges when an individual encounters environmental stimuli or information. These integrated cognitions of the environment, formed by various perceptual systems, ultimately shape the individual’s initial overall impression of the environment. Whether individuals are inclined to seek further interaction with the environment on not hinges on the environment leaves a positive or negative impression. Positive impressions foster pro-environmental thoughts, whereas negative impressions incite resistance to the environment. The quintessential positive emotion is a sense of invitation, suggesting that the environment is amicable, benevolent, supportive, and visually appealing (Ohly et al., 2016), as well as diverse (Rosso et al., 2013).

3. “Preparation” stage—Accessibility

This level mainly corresponds to the pre-contemplation and contemplation stages in the TTM. When the environment is attractive enough, the key factor for elderly individuals to think about whether to go to this place is destination distance as their physical qualification may decline as they age. In other words, accessibility becomes paramount. According to accessibility theory (Cheng et al., 2019; Cascetta et al., 2016), accessibility is defined as the concept that includes activities or destinations and travel impedance (e.g., time, cost, and effort). The more alternatives for reaching destinations or conducting activities and the lower the travel impedance, the higher the level of accessibility.

4. “Action” stage—Walkability

The vestibular system (the sense of balance) and the kinesthetic system (the ability to sense and coordinate movement) are physical indicators affecting the human–environment interaction experience process, especially the walking experience. As elderly people age, their physical strength and coordination stability decline. Therefore, the walkability of the environment is the principal factor and is set as the indicator for this stage.

5. “Maintenance” stage—Safety

After going through the above four stages, individuals may feel satisfied and experience a strong emotional connection to a place, feeling that it is meaningful and significant, and may develop a deep attachment to the place. In this stage, among the most significant or basic environmental factors are safety or security (Lee & Bonaiuto, 2003). Shabak et al. (2015) also hold that psychological disturbances often stem from an absence of place-based support or a stable home base. Hence, safety is prioritized as a key variable, despite its pervasive importance throughout the entire process.

**Figure 1:**
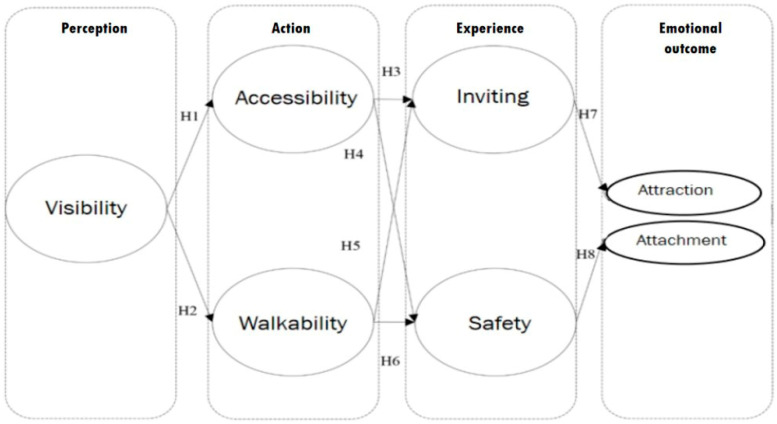
Model assumptions.

The aforementioned five stages and corresponding measurement indicators, along with the two emotional outcome indices of “attraction” and “attachment”, were selected as the configurational indices of the model. Adhering to the logic of the temporal sequence of perception–action–experience–emotion progression and causality, the hypothetical model and assumption are proposed as follows Figure 1:

**H1:**  *Visibility has a positive effect on accessibility.*

**H2:**  *Visibility has a positive effect on walkability.*

**H3:**  *Accessibility has a positive effect on Invitingness.*

**H4:**  *Walkability has a positive effect on Invitingness.*

**H5:**  *Accessibility has a positive effect on safety.*

**H6:**  *Walkability has a positive effect on safety.*

**H7:**  *Invitingness has a positive effect on attraction.*

**H8:**  *Safety has a positive effect on attachment.*

## 3. Methods

### 3.1. Procedure

Initially, a literature review was conducted to identify representative environmental evaluation indices (visibility, Invitingness, accessibility, walkability, safety) that correspond to the stages of change outlined in the TTM theory. Subsequently, these five variables were rearranged according to the stages of the psychological mechanism process, perception–action–experience, to serve as the configurational indices of the hypothetical model’s perceptual component. Moreover, the stages of “attraction” and “attachment” were positioned, respectively, before and after the action as emotional outcome indices. Ultimately, the combination of these two components formed the complete configurational indices that were used to establish the model’s hypotheses. In a Chinese elderly care community, a confirmatory factor analysis of the model was conducted through the distribution of questionnaires to test the hypotheses.

### 3.2. Instruments

Item Generation. Given that the outcome variables are designed to measure “attraction” and “attachment” to facilities, this study employed an adaptive instrument based on the Senior Outdoor Survey (SOS) (Rodiek, 2008); the Place Attachment Scale (PAS) Williams & Roggenbuck (1989), Williams et al. (1992), Williams & Vaske (2003), and its abbreviated version (Boley et al., 2021; Raymond et al., 2010). The basic information section of the questionnaire encompasses age, sex, highest educational qualification, marital status, the number of residents in the dwelling, years of residence at the current address, and the number of diagnosed medical conditions (Lane et al., 2020).

Reliability/Validity Test: To ensure the face validity of the questionnaire, it was reviewed and revised by three experts, achieving a high level of consensus. Post-return of the questionnaires, SPSS 23 and AMOS 20 were utilized for item analysis, reliability, and validity analysis. Cronbach’s α was employed to ascertain the internal consistency of the questionnaire, and composite reliability (CR) was used to replicate reliability. J. F. Hair et al. (2019) have suggested that Cronbach’s α and CR values exceeding 0.7 are deemed acceptable standards.

Questionnaire Adjustment: As the questionnaire items were adapted from previous studies and translated into Chinese, certain settings and expressions may not align with China’s national conditions and cultural context. A pilot study was conducted in Chengdu City to identify and rectify these inadaptations. Three experts include one is an architect, one is a landscape designer, and one is a psychologist from Chengdu Hospital. There are 12 people took part in the pilot study. Take something into consideration, like the time needed to complete the questionnaire, the appropriate order of questions, sufficient space for responses, and clarity of instructions. The following adjustments were made based on their feedback, including the logical structure of questions and the way of inquiry, which improved the effectiveness of the questionnaire. The font size and paragraph interval were adjusted to improve the readability of the questionnaire.

Scoring Method: Following questionnaire testing and pilot study-related adjustments, an adapted questionnaire was formulated, consisting of two parts: the first part includes basic information, such as demographic data, frequency of outdoor activity participation, and duration; the second part encompasses five thematic variables, visibility, invitingness, accessibility, walkability, and safety, with 10 questions per theme, totaling 50 questions for this section. A 5-point Likert scale was applied to rate the degree of agreement for each item, where 1 indicates strong disagreement, and 5 indicates strong agreement.

### 3.3. Participants

#### 3.3.1. Participant Characteristics

Age is a multi-dimensional concept encompassing social age, psychological age, and biological age. Social age is defined by the government as a standard for appellation to facilitate statistical planning. Given the varying population and economic levels across different countries and regions, the age threshold for the elderly also varies. In this study, we focus on the elderly population in China’s retirement communities, adopting the retirement age in China as the benchmark for social age division.

In addition, the elderly can also be categorized based on their physical conditions into active elderly and sedentary elderly (M. Silva et al., 2019). For this study, which examines outdoor activity facilities, the focus is on the active elderly who frequently engage in outdoor activities. We refer to recommendations from the American College of Sports Medicine (ACSM) and the World Health Organization (WHO): middle-aged and older individuals should participate in at least 90–150 min of moderate-intensity physical activity each week (Nelson et al., 2007). Therefore, the inclusion criteria for this study are as follows:

- Men over 60 years old and women over 55 years old.
- Engagement in outdoor activities at least three times a week.
- Each physical activity session lasts 30 min or more.

#### 3.3.2. Ethical Considerations

This study was approved by the ethics committee of the University Putra Malaysia (APPROVAL NO. JKEUPM-2024-834). We affirm that this study adhered to the 1964 Declaration of Helsinki and subsequent amendments. Upholding ethical principles and ensuring respect for participants during the questionnaire collection process, as well as protecting the privacy of study participants, were of utmost importance. Consequently, all participants were guaranteed anonymity and were informed that no sensitive information would be disclosed. They were also informed of their right to withdraw from the study at any time and provided with a clear description of the study’s purpose. We obtained informed consent before beginning the questionnaire.

### 3.4. Data Analysis

The reliability and validity of the tools used in this study are essential to ensure methodological rigor. Prior to conducting structural model validation, we employed statistical software—namely, SPSS 23 and AMOS 20—to perform item analysis and assess reliability and validity, allowing us to ascertain the measurement model’s appropriateness. Subsequently, we confirmed the overall suitability of the study model with acceptable fit indices and then proceeded with model validation. In summary, the reliability and validity of the items and constructs were evaluated through first-order confirmatory factor analysis, followed by structural equation modeling. The analysis within the structural model primarily focuses on the path coefficients between latent variables and hypothesis testing.

## 4. Results

This study employed a combination of purposive sampling and snowball sampling methods to collect data. Part of the data was gathered by distributing questionnaires on community announcement boards. Meanwhile, an online questionnaire was administered via Questionnaire Star (a widely recognized questionnaire platform in China). Distributing questionnaires to targeted retirement homes, we have contacted the manager of the Jin Niu district to recommend which one concentrated on the most ages. We have set up community announcement boards at the entrance of the community and when they come in, our research will invite them to fill up the questionnaire. For somebody who does not have the time to sit down, we will provide the QR code for the online questionnaire so they can fill up online at their convenience. The research commenced on June 1st with a pilot study and concluded on September 1st upon the closure of the online survey link. In total, 403 questionnaires were collected. After data preprocessing, a total of 372 valid questionnaires were obtained. Participants’ characteristics are detailed in the below Table 1.

### 4.1. Reliability and Validity Analysis

Cronbach’s α was used to confirm the internal consistency of the test questionnaire, and composite reliability (CR) was used to replicate the reliability. J. F. Hair et al. (2019) have suggested that Cronbach’s α and CR values above 0.70 are considered acceptable standards. In this study, Cronbach’s α values ranged from 0.92 to 0.94 and CR values ranged from 0.83 to 0.93, which meet the recommended criteria, as shown in Table 2 (Ye et al., 2024).

Convergent validity was determined by factor loading (FL) and average variance extracted (AVE). J. F. Hair et al. (2019) stated that the FL value should be higher than 0.50 and, if it is lower than this value, the question should be removed from the original questionnaire (J. F. Hair et al., 2019; Kenny et al., 2015). All of the questions retained in this study met the criteria suggested by scholars, where the FL values of the configurations ranged from 0.508 to 0.828, as shown in Table 2. J. Hair et al. (2011) suggested that the AVE value should be greater than 0.50 to represent the stringent validity of the structure. In this study, the AVE values ranged from 0.617 to 0.696, which meet the recommended criteria, as shown in Table 2 (Ye et al., 2024).

Awang (2015) indicated that if the AVE root number value of each construct is greater than the Pearson correlation coefficient value of other constructs, this means that the construct has construct differentiation validity. The results of the analysis show that each of the constructs in this study has discriminant validity, as shown in Table 3 (Ye et al., 2024, p. 7519).

### 4.2. Model Fit Analysis

Before performing model verification, the model fit needs to be confirmed (Ye et al., 2024). It is recommended that values of χ^2^/df are less than 5 (J. F. Hair et al., 2019), the RMSEA is less than 0.1, the GFI, AGFI, NFI, NNFI, CFI, IFI, and RFI are greater than 0.80 (Abedi et al., 2015), and the PNFI and PGFI equivalents should be greater than 0.50 (J. F. Hair et al., 2019). Regarding the fit index values for this study, the χ^2^/df values ranged from 1.24 to 1.96, the RMSEA values ranged from 0.026 to 0.052, the GFI values ranged from 0.963 to 0.990, the AGFI values ranged from 0.942 to 0.977, the CFI values ranged from 0.987 to 0.998, and the PCFI values ranged from 0.596 to 0.774, which meet the recommended criteria, as shown in Table 4.

### 4.3. Model Verification

In order to scientifically evaluate the quality of the model. The reasons and significance of each indicator are as follows:

(1) Standardized path coefficient

The path coefficient means the direct relationship between potential variables. It reflects the direction and strength of the influence of one latent variable on another. Similar to the βcoefficients in regression analysis. As shown in Table 5. If the standardized path coefficient is positive, it indicates that variable 1 has a positive influence on variable 2, and otherwise, it has a negative influence.

(2) *p*-value

The significance test is mainly used to determine whether the relationship between variables is significant. When the *p*-value is less than 0.05, the hypothesis passes the test: when the *p*-value is greater than 0.05, the hypothesis fails the test, that is, the hypothesis is not valid. In the calculation result, the “*” is usually used to indicate the range of the *p* value, *** indicating *p* < 0.001. ** indicating *p* < 0.01, * indicating *p* < 0.05.

The model verification results show the following: Hypothesis 1 was supported, as visibility has a positive effect on accessibility (β = 0.773 ***). Hypothesis 2 was supported, as visibility has a positive effect on walkability (β = 0.805 ***). Hypothesis 3 was supported, as accessibility has a positive effect on Invitingness (β = 0.472 **). Hypothesis 4 was supported, as walkability has a positive effect on Invitingness (β = 0.409 ***). Hypothesis 5 was supported, as accessibility positively affects safety (β = 0.404 ***). Hypothesis 6 was supported, as walkability has a positive effect on safety (β = 0.454 ***). Hypothesis 7 was supported: Invitingness positively affects attraction (β = 0.728 ***). Hypothesis 8 was supported, as safety has a positive effect on attachment. (β = 0.692 ***).

(3) R^2^

In structural equation models, in addition to the path coefficient can clarify the strength and direction of the relationship between variables, R^2^ is also an important statistic, which represents the explanatory power, that is, the extent to which the independent variable can explain the dependent variable. Together with the path coefficient, it determines the understanding of the relationship between the variables of the path model.

Standard: the value range between 0 and 1, the greater the value representation model is stronger the explanatory power of the data.

**Figure 2:**
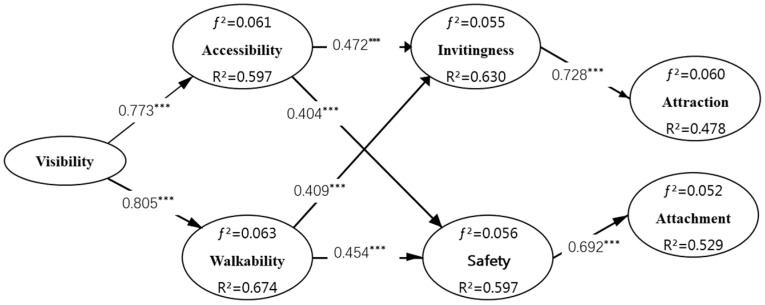
Model verification, *** *p* < 0.001.

When all the indicator calculations and test results have been added to the path diagram. The validated structural model is shown below in Figure 2.

As the date indicator, it might visibility have stronger effects on walkability than accessibility (β1 < β2, f1 < f2). Accessibility has stronger effects on invitingness than safety (β3 > β4, f3 > f4). Walkability has stronger effects on safety than Invitingness (β6 > β5, f6 > f5),

(4) Indirect effect analysis

The results of the indirect effect analysis showed that visibility has an indirect positive effect on safety, Invitingness, attraction, and attachment; walkability also has indirect positive effects on attachment and attraction. Accessibility also has indirect positive effects on attachment and attraction as demonstrated in Table 6.

## 5. Discussion and Conclusions

In summary, this study sought to identify a pathway to sustainable participation through the use of a stage-based model. By Drawing upon previous research findings and application experiences with the TTM theory, a hypothesis model was proposed that incorporates environmental design factors. The model was then verified through confirmatory factor analysis. Consistent with (Zubala et al., 2017) examine the effectiveness of interventions to promote the uptake and maintenance of PA for community-dwelling older adults. The study adopted interventions that typically incorporated behaviour change techniques (BCTs) and examined TTM theory as the analysis theoretical base. The research design, consistent with (Chen & Zhang, 2025) that study based on a questionnaire survey and field measurement data, combined with a data analysis method to systematically discusses the effects of different built environment characteristics on outdoor activity behaviors (frequency and stay time). Theoretically, this research has some similarities to (Lin, 2017, p. 3) which adopts a Western theoretical framework to investigate the community participation behavior of older adults in China. However, this study does not consider the framework component from the perspective of outside physical qualifications, like transport systems, architectural design, and socio-cultural facilities in the built environment, but from the individual introspect psychological process stages. Moreover, consistent with (Ye et al., 2024) that proposed an analysis model based on 5 pillars (variables) contributes to an overall well-being indicator, This study also proposed an analysis model based on 5 pillars (variables), but it contributes to two independent variables, “attraction” and “attachment” which meet the requirements for the occurrence and persistence of behaviors.

Practically, the findings could potentially predict the factors influencing community participation among older adults in China, thus informing government agencies and community service organizations with respect to upgrading community facilities and developing service programs for this demographic in the future (Lin, 2017).

They have policy implications for assisting elderly people’s perception of certain facility interaction issues and for establishing an aging-friendly environment policy targeting this population. For instance, through identifying key environmental factors that influence community participation among older adults and taking them into the urban design plan consideration. For one thing, it can guide the constructor to upgrade existing community facilities and design new ones to meet these requirements, like ensuring proper lighting, well-maintained walkways, and easy-to-use amenities, increasing the availability and quality of green spaces within communities. For another, it can guide to design of spaces that facilitate social interaction among older adults, like creating communal areas, seating, and gathering spots within community facilities.

For policymakers, the findings can help to develop targeted service programs for older adults based on the identified factors that influence their outdoor activity participation. Furthermore. The findings can help to draft and write regulatory documents or safety specifications to help implement safety measures to ensure that community facilities and outdoor spaces are secure and well-maintained so that can minimize safety risks for older adults. Finally, the findings can help them monitor and evaluate the effectiveness of upgraded facilities and service programs in promoting outdoor activity participation among older adults. Use this feedback to make necessary adjustments and improvements for policy-making.

Theoretically, the transtheoretical model (TTM) was selected for this study due to its use of stages of change as the central organizing construct. The results of this study confirm the relevance of the transtheoretical model (TTM) in the context of outdoor activities, specifically the adaptation involved in incorporating affective judgments such as enjoyment, affective attitude, and intrinsic motivation (Rhodes et al., 2021), highlighting its potential to enhance locational attraction and attachment. Although this study used the TTM model to construct a pathway to sustainable outdoor activities, there are more factors that influence participation than just that.This model can be instrumental in developing further stage-matched interventions path tailored to professionals in the various field.

In conclusion, for behavioral application analysis, the human environment interaction (HEI) model offers a holistic and dynamic framework for analyzing behaviors (Marcheschi et al., 2014). Originally defined by Hanson et al. (2013), this concept quantifies the number and diversity of motivator opportunities accessible from a specific location through a comprehensive system.

The research presented here is limited, as it only integrates micro-level individuals and meso-level communities. The participants were recruited only in Chengdu city and limited to a specific age demographic which could induce geographical limitation and Sampling Bias. The geographical limitation is just because the study chose the single case study method and selected the most active and ideal climate and city which ruled out environmental factors and personal physical factors. As for sampling bias. Only the case analysis under the most ideal condition is considered. However, there are many other cases in China and people in other countries who are not in this ideal condition. Therefore, future research needs to take into account the generality of the research results to other cases in other places in China and even around the world. On the other hand, there is self-report bias. Likert scale questionnaire with 5 dimensions was adopted in this study, which has a profound problem: Standard analytic approaches do not control for the confounding effects of idiosyncratic response bias. The research uses techniques such as cross-referencing self-reports with other data sources or implementing consistency checks within the self-report measures to control for response biases.

In future studies, various definitions from different levels and disciplines could be introduced to further refine and expand upon this foundational concept. Future research can strengthen methodology by considering conducting longitudinal studies to track changes over time to help identify trends and patterns that may not be evident in cross-sectional studies. Furthermore, future research can explore incorporating experimental designs of psychological factors including personality traits, cognitive processes, or emotional states that may influence the outcomes of the study. Also, future research can investigate how different behaviors might moderate the effects observed in the study. This could involve looking at lifestyle choices, social behaviors, or other actions that could impact the variables under study.

## Figures and Tables

**Table 1:** Descriptive statistics of elderly respondents.

Variable	Description	Percent (%)
Gender		
	Male	40.8
	Female	59.1
Age (yr)		
	55–59	23.1
	60–69	42.4
	70–79	25.2
	80–89	8.8
	≥90	0.2
Marital Status		
	Single	8.0
	Married	79.8
	Divorced	6.4
	Widowed	5.6
Educational Level		
	Primary/Junior	37.3
	High School	38.9
	Junior College	19.6
	Undergraduate/Bachelor	0.2
Resident Time		
	<1 year	1.0
	1–5 year	8.8
	6–10 year	22.8
	>10 year	67.4

**Table 2:** Reliability and validity analysis.

Construct	M	α	CR	AVE	FL	t
		>0.7	>0.7	>0.5	>0.5	>3
Visibility	3.137	0.944	0.877	0.618	0.601–0.828	12.052–12.627
Invitingness	3.095	0.946	0.893	0.655	0.611–0.699	11.944–12.424
Accessibility	3.130	0.946	0.882	0.629	0.546–0.743	11.844–12.433
Walkability	3.123	0.946	0.934	0.617	0.579–0.746	12.004–12.446
Safety	3.074	0.949	0.907	0.696	0.598–0.761	12.020–12.513
Attraction	3.153	0.921	0.841	0.669	0.562–0.703	10.968–11.692
Attachment	3.106	0.924	0.838	0.664	0.508–0.718	10.049–11.877

**Table 3:** Discriminant validity.

	Visibility	Invitingness	Accessibility	Walkability	Safety	Attraction	Attachment
Visibility	0.788						
Invitingness	0.696	0.798					
Accessibility	0.71	0.688	0.797				
Walkability	0.74	0.676	0.707	0.796			
Safety	0.669	0.647	0.659	0.687	0.805		
Attraction	0.653	0.654	0.659	0.668	0.619	0.812	
Attachment	0.661	0.651	0.677	0.673	0.628	0.589	0.819

**Table 4:** Model fit analysis.

Index	χ^2^	df	χ^2^/df	RMSEA	GFI	AGFI	CFI	PCFI
Threshold value	-	-	<5	<0.1	>0.8	>0.8	>0.8	>0.5
Visibility	54.474	35	1.566	0.039	0.972	0.956	0.987	0.772
Invitingness	60.282	35	1.722	0.044	0.968	0.950	0.991	0.770
Accessibility	55.679	35	1.328	0.030	0.975	0.961	0.996	0.774
Walkability	46.928	35	1.479	0.036	0.975	0.960	0.994	0.773
Safety	68.844	35	1.967	0.051	0.963	0.942	0.988	0.768
Attraction	11.197	9	1.244	0.026	0.990	0.977	0.998	0.599
Attachment	18.188	9	2.021	0.052	0.984	0.963	0.994	0.596

**Table 5:** Summary table of model regression coefficients.

X	→	Y	Unstandardized Path Coefficient	SE	z (CR)	*p*	Standardized Path Coefficient
Visibility	→	Accessibility	0.717	0.037	19.392	0.000	0.773
Visibility	→	Walkability	0.747	0.035	21.263	0.000	0.805
Accessibility	→	Invitingness	0.425	0.040	10.525	0.000	0.472
Accessibility	→	Safety	0.370	0.043	8.663	0.000	0.404
Walkability	→	Invitingness	0.364	0.040	8.989	0.000	0.409
Walkability	→	Safety	0.421	0.043	9.829	0.000	0.454
Invitingness	→	Attraction	0.700	0.042	16.616	0.000	0.728
Safety	→	Attachment	0.652	0.042	15.397	0.000	0.692

**Table 6:** Indirect effects analysis.

Construct	Visibility		Walkability		Accessibility	
	β	95%CI	Β	95%CI	β	95%CI
Safety	0.677 **	[0.589, 0.752]				
Invitingness	0.694 **	[0.604, 0.765]				
Attachment	0.468 **	[0.369, 0.573]	0.314 **	[0.193, 0.442]	0.279 **	[0.161, 0.414]
Attraction	0.505 **	[0.398, 0.599]	0.297 **	[0.16, 0.433]	0.344 **	[0.21, 0.49]

Note: ** *p* < 0.01.

## Data Availability

We do not analyze or generate any datasets, because our work proceeds within a theoretical and mathematical approach. Furthermore, primary and secondary sources and data supporting the findings of this study were all publicly available at the time of submission.

## Abbreviations

The following abbreviations are used in this manuscript:

CCRCs	Continuing care retirement community
HEI	Human-Environment Interaction Model
TTM	The Transtheoretical Model theory
SoC	Stages of change
PC	Processes of change
DB	Decisional balance
SE	Self-efficacy
